# Identification of *Drosophila* centromere associated proteins by quantitative affinity purification-mass spectrometry

**DOI:** 10.1016/j.dib.2015.07.016

**Published:** 2015-07-26

**Authors:** Teresa K. Barth, Georg O.M. Schade, Andreas Schmidt, Irene Vetter, Marc Wirth, Patrick Heun, Axel Imhof, Andreas W. Thomae

**Affiliations:** a Munich Center of Integrated Protein Science and Adolf-Butenandt Institute, Ludwig-Maximilians-University of Munich, 80336 Munich, Germany; b Max-Planck Institute of Immunobiology and Epigenetics, 79108 Freiburg, Germany

**Keywords:** CENP-A, CID, Centromere, Drosophila, Chromatin proteomics

## Abstract

Centromeres of higher eukaryotes are epigenetically defined by the centromere specific histone H3 variant CENP-A^CID^. CENP-A^CID^ builds the foundation for the assembly of a large network of proteins. In contrast to mammalian systems, the protein composition of Drosophila centromeres has not been comprehensively investigated. Here we describe the proteome of *Drosophila melanogaster* centromeres as analyzed by quantitative affinity purification-mass spectrometry (AP-MS). The AP-MS input chromatin material was prepared from *D. melanogaster* cell lines expressing CENP-A^CID^ or H3.3 fused to EGFP as baits. Centromere chromatin enriched proteins were identified based on their relative abundance in CENP-A^CID^–GFP compared to H3.3-GFP or mock affinity-purifications. The analysis yielded 86 proteins specifically enriched in centromere chromatin preparations.

The data accompanying the manuscript on this approach (Barth et al., 2015, Proteomics 14:2167-78, DOI: 10.1002/pmic.201400052) has been deposited to the ProteomeXchange Consortium (http://www.proteomexchange.org) via the PRIDE partner repository with the dataset identifier PXD000758.

**Table:** 

**Specifications Table**	
Subject area	Biology
More specific subject area	Chromatin research, *Drosophila* centromere composition
Type of data	Figures, Table
How data was acquired	Mass spectrometry. LC–MS/MS (LC: Ultimate 3000 HPLC system, Thermo-Fisher Scientific. MS: Orbitrap XL, Thermo-Fisher Scientific)
Data format	Raw (*.raw), MaxQuant output text files (*.zip) and search parameters (*.xml)
Experimental factors	Micrococcal nuclease solubilized chromatin from GFP-fusion H3 variant expressing *D. melanogaster* cell lines analyzed by affinity purification-mass spectrometry.
Experimental features	Micrococcal nuclease solubilization of chromatin, anti-GFP affinity purification, SDS-PAGE, in-gel tryptic digestion, LC–MS/MS analysis of peptides, quantitative comparison of protein enrichment in CENP-A^CID^ vs. H3.3 vs. mock AP-MS samples
Data source location	Munich, Germany
Data accessibility	Uploaded to the ProteomeXchange Consortium webpage. Dataset identifier PXD000758.
	http://proteomecentral.proteomexchange.org/cgi/GetDataset?ID=PXD000758

**Value of the data**

• Analysis of the *Drosophila* centromere proteome
• Identification of uncharacterized potential centromere components in *Drosophila*
• Quantitative analysis of the composition of specific chromatin regions

## 1 Experimental design and data

**Fig. 1:**
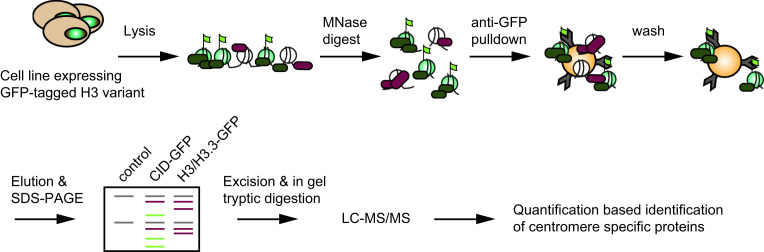
Scheme of experimental strategy. Chromatin of *Drosophila* control cells or cells stably expressing CENP-A^CID^–GFP or H3.3-GFP was digested with MNase and anti-GFP affinity purification was performed on the digested soluble chromatin. After washing steps, proteins were eluted by boiling in Laemmli buffer and separated by SDS-PAGE. Following excision from the gel and in gel tryptic digestion, peptide samples were analyzed by LC–MS/MS. (Adapted from [1].)

**Fig. 2:**
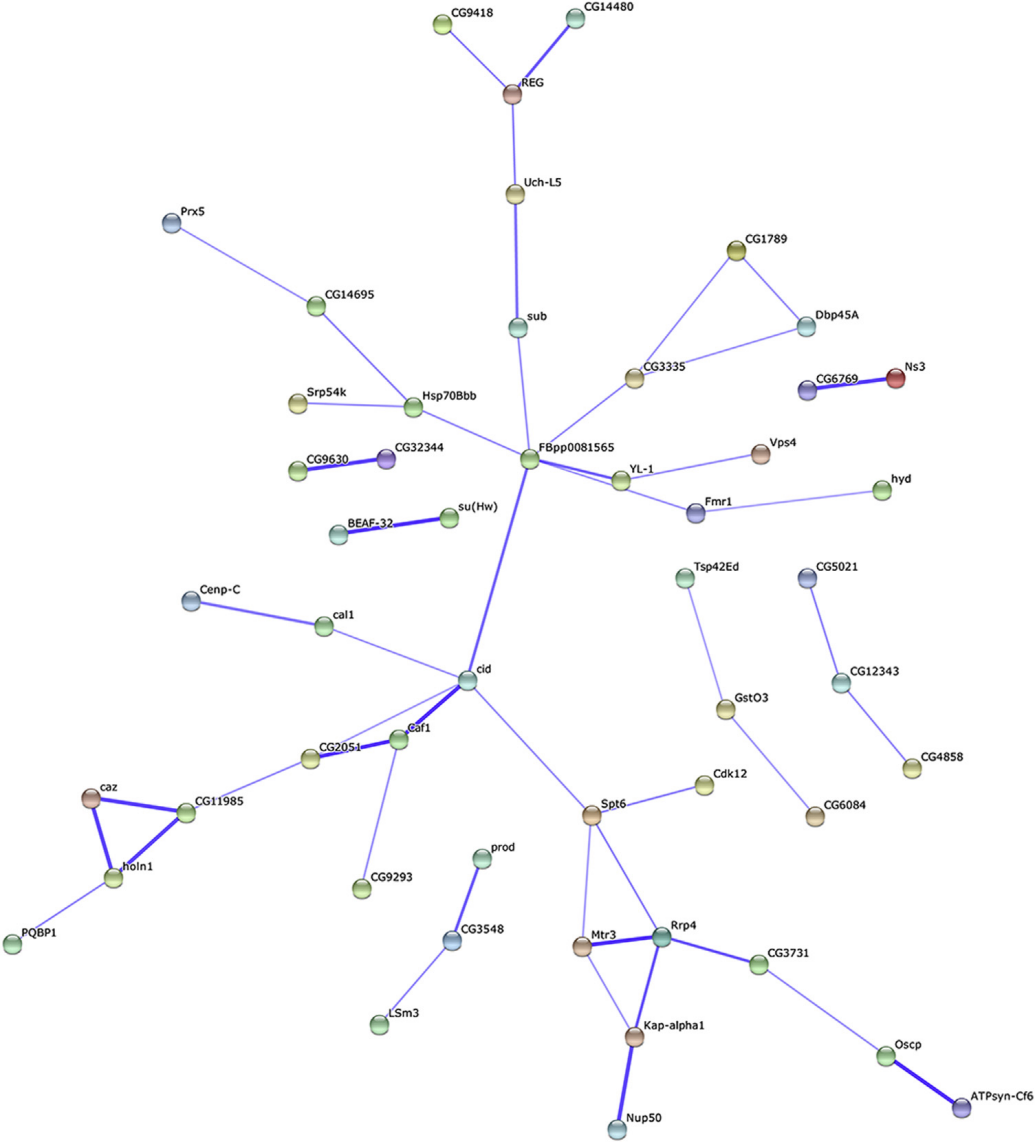
Predicted associations between centromere associated proteins. Confidence view of protein associations among the 86 centromere enriched proteins predicted with the STRING web-tool. Stronger associations are represented by thicker lines.

Fig. 1 illustrates the general workflow of the AP-MS approach (adapted from [1]). In order to discriminate centromere specific proteins from proteins abundantly found in chromatin, we quantitatively compared proteomes isolated from different chromatin regions. To biochemically isolate these different chromatin regions, we made use *Drosophila* cell lines expressing GFP-tagged histone H3 variants as baits. These were either the replacement variant H3.3, which is enriched in euchromatin, or the centromere specific H3 variant CENP-A^CID^. Chromatin from these cells and the parental cell line was isolated and solubilized by Micrococcal Nuclease digestion. This soluble extract served as input material for anti GFP-affinity purification, thereby enriching chromatin fragments together with the associated proteins. Repeated washes were performed to remove unspecifically bound contaminants. The associated proteins were eluted under denaturating conditions and fractionated via SDS-PAGE. After in gel-tryptic digestion, peptides were extracted, concentrated and analyzed by LC–MS/MS. Three independent AP-MS experiments were performed per cell line used. Intensity-based absolute quantification (iBAQ) values from the output of the MaxQuant software package were used as a measure for the abundance of identified proteins. Average iBAQ values were calculated for the different samples and in case the protein was not detected, the iBAQ values were imputed from a random distribution (see Section 2.5). Centromere enrichment was calculated by dividing the average iBAQ value for each protein in the CENP-A^CID^–GFP purification by the corresponding iBAQ value in the chromatin purification from untransfected parental or H3.3-GFP expressing cell lines, respectively. A factor was considered centromere specific if its log2-fold enrichment over both controls was more than four. Using these criteria, we identified 86 proteins that were specifically enriched in CENP-A^CID^–GFP containing chromatin (Table 1 and [1]). Known CENP-A^CID^-binding proteins such as Cal1, the centromeric proteins CENP-C, or CAF-1 were also found enriched in CENP-A^CID^ chromatin, demonstrating the general applicability of the technique to detect proteins enriched in centromeric chromatin [2]. While centromere association of most of the 86 identified proteins has not been reported so far, several associations among the proteins are predicted using the “Search Tool for the Retrieval of Interacting Genes/Proteins” (STRING) (Fig. 2) [3]. This indicates that a complex network of interactions contributes to centromere function or maintenance.

## 2 Materials and methods

### 2.1 Cell culture

The *Drosophila* Schneider Line 2 derived L2–4 cell line was used for all experiments. Cells were maintained at 25 °C in *Drosophila* Schneider medium supplemented with 10% fetal calf serum and penicillin/streptomycin. Stable cell lines were established by XtremeGENE HP mediated transfection of GFP-fusion expression constructs following four weeks of Hygromycin B selection (100 µg/mL).

### 2.2 Chromatin preparation for AP-MS

Asynchronously growing cells were harvested by centrifugation and washed in PBS. Cells were resuspended in ice-cold hypotonic buffer (10 mM HEPES, pH 7.6; 15 mM NaCl; 1.5 mM MgCl_2_; 0.1 mM DTT; freshly added protease inhibitors: PMSF, Aprotinin, Leupeptin, Pepstatin) and lysed for 10 min on ice by adding Triton X-100 to a final concentration of 0.1%. Nuclei were pelleted by centrifugation, washed with PBS and chromatin was solubilized for 10 min at 26 °C by micrococcal nuclease (MNase) digestion in EX100 buffer (10 mM HEPES, pH 7.6; 100 mM NaCl; 1.5 mM MgCl_2_; 0.5 mM EGTA; 2 mM CaCl_2_; 10% glycerol (v/v); freshly added protease inhibitors) containing 2000 U MNase per one billion nuclei. Chromatin was released by increasing the sodium chloride concentration to 300 mM and applying ten strokes in a Dounce homogenizer with a tight-fit pestle. Following one hour incubation at 4 °C, insoluble material was pelleted for 20 min at 20,000*g* and the supernatant was precleared with Protein A Sepharose beads yielding the AP-MS input extract.

### 2.3 Affinity purification and sample preparation for mass spectrometry

GFP-Trap agarose beads (ChromoTek) were used as affinity resin. The beads were preblocked in 0.5% BSA, 0.5% polyvinylpyrrolidone dissolved in EX100 buffer by over-head rotation for 30 min. The input extract was added to the blocked beads and affinity purification was performed for 2 h at 4 °C on an over-head rotator. The affinity resin with bound complexes was washed three times for 5 min at 4 °C with EX300 buffer and bound proteins were eluted by boiling beads in Laemmli buffer for 10 min at 95 °C. Eluted proteins were separated by SDS-PAGE using a 15% polyacrylamide gel and the gel was stained with Coomassie Brilliant Blue G-250. Whole lanes were excised from the gel with a disposable gridcutter (Gel Company) and split into eight vials. Following destaining, reduction of disulfide bonds with dithiothreitol and alkylation with iodoacetamide, in-gel tryptic digestion was performed. Resulting peptides were collected by acid extraction of the gel pieces, concentrated by evaporation, and resuspended in 0.1% TFA.

### 2.4 LC–MS/MS

Peptides were injected into an Ultimate 3000 HPLC system (Thermo-Fisher Scientific). Samples were desalted online by a C18 micro-column (5 mm×300 µm id 5 mm, packed with C18 PepMapTM, 5 µm, 100 Å, Thermo-Fisher Scientific), and peptides were separated with a gradient from 5% to 60% acetonitrile in 0.1% formic acid over 40 min at 300 nL/min on a C18 analytical column (10 cm×75 µm, packed in house with C18 PepMapTM, 3 µm, 100 Å, Thermo-Fisher Scientific). The effluent from the HPLC was directly infused into the LTQ Orbitrap mass spectrometer (Thermo-Fisher Scientific) via a nano-electrospray ion source. The MS instrument was operated in the data-dependent mode to automatically switch between full-scan MS and MS/MS acquisition. Survey fullscan MS spectra (*m/z* 350–2000) were acquired in the Orbitrap with resolution 60,000 at *m/z* 400. For all measurements with the Orbitrap detector, three lock-mass ions from ambient air (*m*/*z*=371.10123, 445.12002, 519.13882) were used for internal calibration as described [4]. The six most intense peptide signals with charge states between two and five were sequentially isolated applying a 1 Da window centered around the most abundant isotope to a target value of 10,000 and fragmented in the linear ion trap by collision-induced dissociation. Fragment ion spectra were recorded in the linear trap of the instrument. Typical mass spectrometric conditions were as follows: spray voltage, 1.4 kV; no sheath and auxiliary gas flow; heated capillary temperature, 200 °C; activation time, 30 ms; and normalized collision energy, 35% for collision-induced dissociation in linear ion trap.

### 2.5 Protein identification and statistical analysis

For protein identification, the raw data were analyzed with the Andromeda algorithm of the MaxQuant protein analysis package (version 1.2.2.5) against the Flybase dmel-all-translation-r5.24.fasta database including reverse sequences and contaminants. The Trypsin/P enzyme was selected, allowing for maximum two missed cleavages. Carbamidomethylation of cysteine was set as fixed modification; methionine oxidation and protein *N*-acetylation were included as variable modifications. The mass tolerance of the initial search was 20 ppm; after recalibration, a 6 ppm mass error was applied for the main search. Fragment ions were searched with a mass offset of 0.5 Da using the six most intense signals per 100 Da. Searching for secondary peptide hits within already assigned MS/MS spectra was enabled. The search results were filtered with a peptide and protein false discovery rate of 0.01 with a minimum peptide length of six amino acids. Protein identifications with at least one unique peptide were accepted. For quantification, the intensity-based absolute quantification (iBAQ) values were calculated from peptide intensities and the protein sequence information [5] of unmodified, M/oxidated, and acetylated peptide species with a minimum of two peptides per protein.

As preparation for statistical analysis, protein hits representing reversed sequences or contaminants and protein hits without quantification values were removed from the list of identified proteins from three biological replicates. iBAQ quantification values were log2-transformed and subsequently missing values were imputed from a random distribution centered at 1/3×log2 of the obtained experimental data. The imputation was repeated three times to reduce effects of the random value distribution. ANOVA was applied in DanteR (vs 0.2, PNNL, Richland, WA, USA) [6] to calculate protein enrichment factors and *p*-values and obtained *p*-values were corrected for multiple hypothesis testing by the Benjamini–Hochberg method [7].

### 2.6 STRING analysis

Protein names from Table 1 were subjected to STRING analysis using the web-tool available via http://string-db.org/ [3]. Fig. 2 shows the confidence view with the active prediction methods “Experiments”, “Databases” and “Textmining” and medium confidence score (0.4).

## Figures and Tables

**Table 1:** 86 Proteins specifically associate with centromere chromatin List of 86 CENP-A^CID^ chromatin co-purifying proteins ranked according to their calculated enrichment over mock purification.

Name	FBpp	FBgn	Uniprot	CID-GFP/control		CID-GFP/H3.3-GFP	
				log2	*P*-value	log2	*P*-value

cid	FBpp0086787	FBgn0040477	Q9V6Q2	15.9±1.1E−01	1.60E−02	11.1±4.5E−05	5.80E−02
CG14480	FBpp0086006	FBgn0034242	Q7JWU9	11.8±8.7E−02	1.70E−05	11.6±7.4E−02	1.40E−05
cal1	FBpp0082841	FBgn0038478	Q9VEN2	11.3±9.1E−02	1.60E−05	11.2±7.6E−02	1.70E−05
CG2051	FBpp0078319	FBgn0037376	Q0KIB3	10.8±1.0E−05	5.40E−02	8.3±3.1E−07	1.10E−01
CG13117	FBpp0079460	FBgn0032140	Q9VL93	9.2±1.1E−01	7.60E−04	9.6±2.6E−02	6.50E−04
CG34191	FBpp0111299	FBgn0085220	A8DYH2	9.2±1.8E−01	3.50E−04	9.5±1.3E−01	3.00E−04
CG6769	FBpp0074267	FBgn0030878	Q9VX08	8.6±9.2E−02	2.20E−04	8.4±7.2E−02	2.60E−04
Vps4	FBpp0074278	FBgn0027605	Q9Y162	8.0±4.3E−01	9.20E−02	7.5±4.0E−01	1.00E−01
CG4972	FBpp0079578	FBgn0032217	Q9VKZ7	7.7±1.6E−01	6.20E−05	7.6±4.7E−02	5.30E−05
Caf1	FBpp0291059	FBgn0263979	E1JIL4	7.6±8.6E−03	1.90E−04	7.5±1.1E−02	1.70E−04
LSm3	FBpp0083893	FBgn0051184	Q8IMX8	7.5±7.6E−02	3.90E−04	7.7±5.4E−02	2.50E−04
Kap-alpha1	FBpp0074650	FBgn0024889	O76521	7.5±4.4E−01	1.10E−02	8.3±2.5E−01	4.60E−03
REG	FBpp0073561	FBgn0029133	Q9V3P3	7.3±1.1E−04	4.00E−01	13.4±3.8E−02	6.40E−02
ifc	FBpp0078810	FBgn0001941	Q94515	7.3±9.8E−02	2.20E−04	7.1±6.8E−02	2.60E−04
CG11985	FBpp0081484	FBgn0040534	Q9VHI4	7.2±7.5E−02	7.10E−04	7.5±6.7E−02	3.50E−04
CG9293	FBpp0080103	FBgn0032516	Q9VJY8	7.1±1.7E−01	3.70E−05	7.1±6.2E−02	4.30E−05
CG8891	FBpp0078684	FBgn0031663	Q9VMW7	7.1±1.2E−01	7.70E−03	7.4±1.2E−01	5.00E−03
CG3548	FBpp0072222	FBgn0035033	Q9W140	7.0±1.0E−01	2.00E−04	7.0±4.9E−02	2.50E−04
Hsp70Bbb	FBpp0082105	FBgn0051354	Q9VG58	7.0 ±2.1E−02	1.40E−03	6.9±6.0E−02	1.50E−03
SRPK	FBpp0086515	FBgn0026370	Q0E965	7.0±6.1E−01	2.60E−02	7.6±2.8E−01	1.40E−02
Unc-76	FBpp0070360	FBgn0040395	Q9W525	7.0±9.5E−02	8.80E−03	6.3±8.1E−02	1.20E−02
GstO3	FBpp0076348	FBgn0035904	Q9VSL2	6.9±2.1E−01	8.40E−02	7.8±9.1E−02	4.30E−02
Srp54k	FBpp0076872	FBgn0010747	Q9V3D9	6.9±5.0E−01	1.90E−01	6.4±5.3E−01	1.30E−01
CG32069	FBpp0075970	FBgn0052069	Q9VTE1	6.7±1.7E−01	1.60E−04	6.6±6.5E−02	1.70E−04
alphaTub85E	FBpp0081565	FBgn0003886	P06604	6.7±7.2E−02	1.80E−03	7.1±1.0E−01	9.20E−04
MED30	FBpp0072457	FBgn0035149	Q9W0P3	6.6±7.5E−02	1.40E−04	6.6±6.6E−02	1.80E−04
Fwe	FBpp0075297	FBgn0261722	Q95T12	6.6±9.6E−02	1.60E−04	6.4±8.1E−02	1.30E−04
Rrp4	FBpp0072020	FBgn0034879	Q9W1M9	6.4±1.1E−01	1.70E−03	6.3±6.4E−02	1.30E−03
CG1789	FBpp0071194	FBgn0030063	Q9W3C0	6.3±2.1E−02	1.10E−03	6.8±2.7E−02	7.30E−04
qkr58E-2	FBpp0071739	FBgn0022985	Q9W254	6.3±9.7E−02	1.80E−04	6.1±6.8E−02	2.10E−04
prod	FBpp0085785	FBgn0014269	Q7JNE1	6.3±1.4E−01	1.20E−04	6.2±1.1E−01	1.50E−04
Tsp42Ed	FBpp0085513	FBgn0029507	Q7JWV7	6.3±1.0E−01	2.00E−04	6.1±8.1E−02	1.90E−04
CG6180	FBpp0079979	FBgn0032453	Q9VK60	6.3±1.4E−01	9.20E−04	6.6±2.8E−02	6.70E−04
CG7945	FBpp0075353	FBgn0036505	Q95RY2	6.3±4.2E−01	1.80E−01	5.1±1.0E+00	2.80E−01
CG14695	FBpp0081811	FBgn0037850	Q9VGV0	6.2±1.9E−01	2.30E−03	6.5±1.1E−01	1.80E−03
UQCR-C1	FBpp0082459	FBgn0038271	Q9VFF0	6.2±6.5E−02	4.00E−04	6.0±5.0E−02	5.00E−04
ATPsynCF6	FBpp0083824	FBgn0016119	Q24407	6.2±2.2E−01	1.20E−05	6.2±5.0E−02	1.30E−05
BEAF-32	FBpp0086571	FBgn0015602	Q7JN06	6.1±5.9E−02	5.30E−04	6.2±7.5E−02	5.70E−04
CG5021	FBpp0290362	FBgn0035944	Q8IQC1	6.1±6.2E−01	1.90E−01	4.9±1.2E+00	3.10E−01
PH4alphaEFB	FBpp0085012	FBgn0039776	Q9VA69	6.0±1.0E−01	1.70E−03	6.3±9.6E−02	1.50E−03
Ns3	FBpp0070284	FBgn0266284	Q9W590	6.0±9.2E−02	2.60E−01	6.0±7.1E−02	2.10E−01
CG1265	FBpp0073084	FBgn0035517	Q9VZF3	6.0±1.5E−01	9.70E−04	6.0±5.0E−02	1.10E−03
CG12343	FBpp0087342	FBgn0033556	Q9V5Q4	6.0±6.5E−02	1.20E−04	6.0±6.7E−02	1.10E−04
CG11030	FBpp0078766	FBgn0031736	Q9VMM7	5.9±3.8E−01	3.00E−01	5.6±4.3E−01	2.40E−01
Hmg-2	FBpp0071501	FBgn0026582	Q9W2K8	5.9±8.6E−02	1.80E−04	5.7±9.0E−02	2.50E−04
Prx5	FBpp0100079	FBgn0038570	Q960M4	5.9±1.2E−01	2.00E−02	6.1±4.6E−02	1.60E−02
Jheh2	FBpp0088908	FBgn0034405	Q7KB18	5.9±6.5E−02	8.40E−04	5.9±1.1E−02	8.20E−04
Pcd	FBpp0084824	FBgn0024841	O76454	5.9±6.6E−02	4.70E−03	6.3±1.1E−01	3.50E−03
caz	FBpp0073996	FBgn0011571	Q27294	5.7±1.3E−01	2.10E−05	6.0±6.1E−02	8.70E−06
Sgf29	FBpp0071543	FBgn0050390	Q9W2I4	5.7±1.9E−01	1.80E−04	5.6±8.5E−02	2.40E−04
vnc	FBpp0076077	FBgn0263251	Q9VT75	5.6±4.8E−02	1.80E−03	6.0±1.4E−01	1.40E−03
Dbp45A	FBpp0087645	FBgn0010220	Q07886	5.6±2.3E−01	1.30E−03	6.0±3.1E−02	1.00E−03
Uch-L5	FBpp0076200	FBgn0011327	Q9XZ61	5.5±1.4E−01	7.70E−04	5.7±6.9E−02	6.00E−04
Fmr1	FBpp0081675	FBgn0028734	Q9NFU0	5.5E±4.5E−02	2.00E−01	5.4±1.2E−01	1.70E−01
gfzf	FBpp0290855	FBgn0250732	Q6NP69	5.5±9.0E−02	2.70E−04	5.5±1.1E−01	2.50E−04
su(Hw)	FBpp0082404	FBgn0003567	P08970	5.4±8.7E−02	6.00E−04	5.3±8.2E−02	4.10E−04
CG6084	FBpp0075870	FBgn0086254	Q9VTK9	5.4±9.2E−02	7.80E−03	5.6±4.8E−02	6.70E−03
Nup50	FBpp0087861	FBgn0033264	Q7K0D8	5.4±2.8E−02	1.10E−01	4.1±3.3E−01	2.10E−01
RagC-D	FBpp0087883	FBgn0033272	Q7K519	5.4±1.5E−01	5.40E−04	5.3±1.6E−01	4.00E−04
CG17271	FBpp0083397	FBgn0038829	Q9VDI5	5.4±6.9E−01	2.50E−01	4.3±1.2E+00	1.80E−01
Caf1	FBpp0082511	FBgn0263979	Q24572	5.3±1.7E−07	7.20E−03	5.3±6.8E−07	6.50E−03
CG1091	FBpp0081154	FBgn0037470	Q9VI58	5.3±1.2E−01	7.20E−04	5.3±1.3E−01	9.30E−04
bor	FBpp0082728	FBgn0040237	Q9VEX6	5.1±1.2E−01	2.80E−02	4.2±4.1E−01	6.80E−02
Spt6	FBpp0070861	FBgn0028982	Q9W420	5.1±3.9E−02	1.90E−01	4.8±7.6E−02	1.90E−01
YT521-B	FBpp0072940	FBgn0027616	Q9VZQ1	5.0±3.5E−01	3.50E−01	5.0±9.7E−02	3.20E−01
CG5482	FBpp0085842	FBgn0034368	Q7K3D4	5.0±9.0E−02	3.40E−04	5.3±5.3E−02	2.20E−04
CG1309	FBpp0073085	FBgn0035519	Q9VZF1	4.9±7.4E−03	8.90E−03	5.1±3.7E−02	4.00E−03
hyd	FBpp0081568	FBgn0002431	P51592	4.9±1.6E−01	1.80E−03	4.8±4.4E−02	2.30E−03
CG32344	FBpp0072475	FBgn0052344	Q8SY39	4.9±5.8E−06	4.20E−01	11.1±4.1E−02	3.10E−02
PQBP1	FBpp0084140	FBgn0039270	Q9VBY6	4.9±2.9E−01	6.90E−02	5.8±2.5E−01	3.30E−02
ATPsynO	FBpp0082522	FBgn0016691	Q24439	4.8±1.0E−01	2.30E−02	5.1±7.6E−02	1.90E−02
CG4858	FBpp0077868	FBgn0037011	Q9VPD2	4.8±1.5E−01	6.70E−01	6.5±5.8E−02	3.30E−01
CG3335	FBpp0076112	FBgn0036018	Q9VT19	4.8±9.8E−02	5.80E−03	5.0±7.8E−02	4.20E−03
CG11076	FBpp0088285	FBgn0039929	Q9V493	4.8±4.3E−02	2.30E−03	4.7±1.0E−01	2.10E−03
YL-1	FBpp0079735	FBgn0032321	Q9VKM6	4.7±8.9E−02	6.90E−04	4.7±5.9E−02	7.10E−04
sub	FBpp0086041	FBgn0003545	Q9V877	4.6±1.0E−01	5.30E−03	4.6±1.3E−01	6.70E−03
Rbcn-3A	FBpp0292404	FBgn0023458	Q9W425	4.6±1.4E−01	3.60E−04	4.5±7.8E−02	4.10E−04
eIF-4B	FBpp0112403	FBgn0020660	Q7PLL3	4.6±8.0E−02	1.90E−02	4.8±8.2E−02	1.30E−02
Pgi	FBpp0087760	FBgn0003074	P52029	4.5±9.7E−02	9.70E−03	4.7±1.6E−02	7.20E−03
CG9630	FBpp0081355	FBgn0037561	Q9VHU1	4.5±7.3E−02	2.80E−03	4.8±1.6E−01	1.20E−03
Cenp-C	FBpp0088911	FBgn0266916	Q9VHP9	4.5±1.3E−01	3.00E−03	4.4±7.0E−02	2.90E−03
Mtr3	FBpp0074687	FBgn0036916	Q9VW53	4.5±1.1E−01	5.20E−01	5.8±6.4E−02	2.70E−01
CG2943	FBpp0081262	FBgn0037530	Q9VHY6	4.5±7.7E−02	6.10E−01	4.4±5.5E−02	5.10E−01
Cdk12	FBpp0078013	FBgn0037093	Q9VP22	4.4±2.2E−01	5.00E−04	4.2±7.5E−02	5.70E−04
CG7518	FBpp0297366	FBgn0038108	A0A0B4K6G6	4.3±1.2E−01	5.40E−04	4.4±7.4E−02	6.00E−04
holn1	FBpp0079616	FBgn0032250	Q9VKV5	4.2±3.8E−02	3.20E−02	4.6±1.2E−01	1.10E−02
